# Circular genomes of two bacterial strains capable of growing in a CO_2_-containing atmosphere

**DOI:** 10.1128/MRA.00685-23

**Published:** 2023-11-29

**Authors:** David Heidler von Heilborn, Alexander Bartholomäus, André Lipski

**Affiliations:** 1 Institute of Nutritional and Food Science, Food Microbiology and Hygiene, University of Bonn, Bonn, Germany; 2 GFZ German Research Centre for Geosciences, Section Geomicrobiology, Potsdam, Germany; Indiana University, Bloomington, Indiana, USA

**Keywords:** modified atmosphere packaging, capnotolerance, food microbiology, taxonomy, carbon dioxide

## Abstract

The bacterial strains *Brochothrix thermosphacta* DH-B18 and *Rathayibacter* sp. DH-RSZ4 were isolated from raw sausage and escalope samples and grown in a CO_2_-rich modified atmosphere. Here, we present both circular genomes obtained by nanopore sequencing.

## ANNOUNCEMENT

Modified atmosphere packaging is used in the food industry for a variety of reasons, including the prevention of microbial spoilage and sensory changes, thus extending the shelf life of several food products. In most cases, carbon dioxide is a major component of these atmospheres and is used for its antimicrobial properties (1).

The Gram-positive bacterial strains *Brochothrix thermosphacta* DH-B18 and *Rathayibacter* sp. DH-RSZ4 were isolated from raw sausage and escalope samples, respectively, by cutting and homogenizing 10 g of sample material with 90 mL Ringer’s solution (Merck, Germany) and plating a suspension of 100 µL on tryptic soy agar (TSA), as described earlier (2). The strains were cultivated on TSA at 10 and 30°C. The mentioned samples of origin were stored and packed under high-CO_2_-containing modified atmospheres before, and the isolates were tested for growth at 20% CO_2_ and 80% O_2_, as previously done (2). Both strains were selected due to their presence in food samples and their capnotolerance and psychrotolerance. The species *Brochothrix thermosphacta* is known for being a food spoilage organism and growing in the presence of elevated CO_2_ levels (3). The genus *Rathayibacter* sp. has not yet been associated with food samples in general, but some species have been isolated from (cultivated) plants, such as onion, and linked to plant diseases (4).

High-molecular-weight DNA was extracted from a culture, which was grown from a single colony, using the Monarch HMW DNA Extraction Kit (NEB BioLabs GmbH, Germany) without specific size selection, and sequencing libraries were prepared using the rapid sequencing kit SQK-RAD004 (Oxford Nanopore Technologies [ONT], Oxford, UK). The sequencing libraries were cleaned using AMPureXP beads (Beckman Coulter, Pasadena, CA, USA) and sequenced using the MinION platform and the Flongle flow cell (ONT). The sequencing reaction ran for 72 h, and the quality was monitored using the MinKNOW interface v22.05.5 (ONT). Default parameters were used for all software unless otherwise specified. Raw sequencing data were base called and demultiplexed with super high accuracy using Guppy v6.0.6 (ONT), resulting in 26,626 reads with an N50 of 16,085 for DH-B18 and 8,998 reads with an N50 of 24,726 for DH-RSZ4. Assembly, first polishing, and circularity assessment were performed with Flye v2.9.1 (5) (parameter –meta –nano-raw). The second polishing was performed using Medaka v1.7.2 (ONT). The genome quality was assessed, and full-length 16S rRNA sequences were recovered using CheckM v1.2.1 (6). Genome characteristics were assessed using QUAST v5.2.0 (7).

The resulting genomes were circular contigs of 2,557,095 and 3,890,578 bp length, including a GC content of 36.5% and 71.5% and coverage of 62× and 23× for DH-B18 and DH-RSZ4, respectively. Plasmids were not detected. By using CheckM v1.2.1 (6), the DH-B18 genome was found to be 99.31% complete and 0.0% contaminated, and DH-RSZ4 was 85.6% complete and 2.2% contaminated. The taxonomic affiliations were inferred using GTDB-Tk v2.1.0 (8), revealing the type strain *Brochothrix thermosphacta* DSM 20171^T^ to be very closely related (ANI 98.98, GCF_000620985.1) to strain DH-B18, and the type strain *Rathayibacter oskolensis* VKM Ac-2121^T^ (ANI 88.61, GCF_900177245.1) represents the closest known species to strain DH-RSZ4. Additionally, the TYGS tool (accessed on 24 July 2023) (9) confirmed the results, with strain DH-B18 having a high 91.9% digital DNA-DNA hybridization (dDDH) value to *B. thermosphacta* DSM 20171^T^, while DH-RSZ4 has only 32.4% dDDH to *R. oskolensis* VKM Ac-2121^T^ as the closest related type strain. The genomes were annotated by National Center for Biotechnology Information using the PGAP pipeline v6.3 (10).

## Data Availability

Genome and raw reads are available at GenBank. The data for *Brochothrix thermosphacta* DH-B18 have BioProject accession number PRJNA993613, genome accession number CP129978, and raw read accession number SRR25232469. The data for *Rathayibacter* sp. DH-RSZ4 have BioProject accession number PRJNA993609, genome accession number CP129977, and raw read accession number SRR25232475.
